# Academic resilience from school entry to third grade: Child, parenting, and school factors associated with closing competency gaps

**DOI:** 10.1371/journal.pone.0277551

**Published:** 2022-11-30

**Authors:** Kate E. Williams, Donna Berthelsen, Kristin R. Laurens

**Affiliations:** 1 Queensland University of Technology (QUT), Centre for Child and Family Studies, Kelvin Grove, Qld, Australia; 2 Queensland University of Technology (QUT), School of Early Childhood and Inclusive Education, Kelvin Grove, Qld, Australia; 3 Queensland University of Technology (QUT), Centre for Child Health and Well-being, Brisbane, Qld, Australia; 4 Queensland University of Technology (QUT), School of Psychology and Counselling, Kelvin Grove, Qld, Australia; 5 Queensland University of Technology (QUT), Centre for Inclusive Education (C4IE), Kelvin Grove, Qld, Australia; King Fahd University of Petroleum & Minerals, SAUDI ARABIA

## Abstract

There is substantial evidence confirming that children who begin school with strong developmental skills tend to maintain positive academic trajectories across the elementary school years. Much less is known about children who begin school with poorer developmental competencies yet go on to achieve academically on par with, or above, their initially more competent peers, demonstrating academic resilience. This study used a large population dataset, the *Longitudinal Study of Australian Children* (n = 2,118) to identify children who demonstrated academic resilience between school entry and third grade, and the child, parenting, and school characteristics associated with this resilience. Findings show that two in five children who were initially identified as academically vulnerable on a school entry measure of language and cognitive skills were classified as academically resilient by Grade 3. In multivariate analysis, higher attentional regulation and receptive vocabulary skills were key factors associated with academic resilience in reading and numeracy, along with paternal consistency (for reading resilience) and fewer sleep problems (for numeracy resilience). Bivariate relations (ANOVAs) showed that resilient children, when compared to children who remained vulnerable, also showed fewer peer problems, fewer behavioral sleep problems, higher levels of parenting consistency and lower levels of parenting anger by mothers and fathers, higher levels of parental engagement in children’s school, and higher levels of teacher self-efficacy. Supporting resilient pathways for children who are identified as vulnerable at school entry should include a particular focus on vocabulary development and attentional regulation, along with social skills and peer relationships, school-based parental engagement, and positive parenting support.

## 1. Introduction

An important enduring policy concern across international contexts is enhancement of educational attainment and engagement in learning across the lifespan [1]. There is now well-established evidence that, at a population level, educational trajectories are largely plotted from a very young age, and heavily influenced by early home learning environments, familial contexts, and exposure to risk factors, including poverty, in the first five years of life [2]. Starting school is an important milestone, and children’s developmental competencies at this time, particularly in relation to language and cognitive skills, are highly correlated with later academic achievement [3–5]. During the early school years, some children who enter school with poorer skills will struggle to meet academic expectations, while others will find a positive educational pathway. This study uses a large longitudinal population dataset to understand which children achieve well academically at school despite early developmental vulnerability (here, we term this pathway academic resilience). Further, we identify which child, parenting, and school factors differentiate these academically resilient children from those children who continue on a lower achievement trajectory. The identification of factors that distinguish students who are resilient despite the odds can be the basis for the design of early intervention programs that enable pathways to academic resilience.

### 1.1. Academic resilience in context

Individuals who succeed developmentally despite exposure to high levels of risk are described as resilient [6]. While socio-demographic differences consistently explain a large portion of the gap between high and low achieving students over time, it is recognised that many students overcome significant developmental risks and go on to have a successful school career [7], with a range of individual and contextual factors likely contributing to this pathway. For example, in a recent study from the United States, a quarter of students entering school with developmental vulnerability achieved competence on mathematics and English assessments by Grade 3 [4]. What is it that distinguishes these children who close this competency gap from those who remain on a low attainment trajectory?

This study is informed by the bioecological model of development which describes how proximal processes operating within and across family and school environments impact children’s outcomes [8]. Within family contexts, responsive and sensitive parenting, early oral language environments, and home learning engagement support children’s early vocabulary and literacy development [9] and numeracy skills [10]. At school, children experience unique teacher characteristics, classroom environments and practices, peer interactions, and school level policies that are heterogeneous in nature across school contexts and, thus, challenging to unpack in assessing influences on child outcomes [11]. Nonetheless, these early school experiences significantly influence children’s academic trajectories. In line with the bioecological model, research identifies three main categories of factors that are likely to influence academic resilience: individual child, parenting, and school factors.

### 1.2. Individual child factors, academic outcomes, and resilience

Extensive evidence exists for the individual skills, developed within surrounding environments, that promote early and ongoing school success. Early knowledge and language-based skills including vocabulary [12], along with social-emotional competencies such as self-regulation [13] and social skills with peers [14], have been consistently identified as key predictors of ongoing academic trajectories. Over the last two decades, young children’s sleep routines, sleep onset, and duration have also emerged as contributors to early cognitive and academic skills [15] with persistent sleep problems associated with risk for poor academic performance across elementary school [16].

Beyond direct prediction of later academic achievement, a range of studies suggest that these same individual child factors are likely to play a moderating role in terms of the relation between early risk and achievement. For example, in a representative national US study (N > 9,000), children’s strong social-emotional-behavioral skills, as reported by teachers, reduced the negative impact of low socio-economic status on children’s reading and mathematics achievement [17]. Similarly increased night-time sleep duration has been found to provide some protection against poorer achievement associated with househould chaos (typified by crowding, disorganisation, and elevated noise levels) [18]. When early risk is conceptualised using developmental skills rather than socio-economic status, similar findings emerge, with high peer competence moderating the association between self-regulation and mathematics achievement [19], and sleep duration moderating relations among reactive temperament and achievement [18]. Taken together, it is apparent that for children who enter school with socio-economic or early developmental risks, some protection in terms of later achievement is offered by stronger cognitive skills (e.g., language skills), non-cognitive skills (e.g., attentional regulation) and healthy sleep.

### 1.3. Parenting, academic outcomes, and resilience

Multiple meta-analyses identify that more positive parental styles including warmth/responsiveness, autonomy support, behavioral control, and authoritative parenting have small positive associations with children’s academic achievement at school [20,21]. More negative parenting qualities including harsh parenting or psychological control, and authoritarian, permissive, and neglectful parenting show small/very small negative associations with academic competence [20,21]. Despite under-representation of fathers in research examining parenting-academic achievement associations, a meta-analysis by Vasquez and colleagues [21] indicates stronger relations when autonomy support is reflective of *both* parents rather than mothers or fathers alone. Data from the NICHD Study of Early Child Care and Youth Development in the United States (N = 723) demonstrated that fathers’ supportive parenting had larger (buffering) effects on their children’s academic achievement in kindergarten and first grade in the context of lower levels of maternal supportiveness, whereas mothers’ supportiveness was associated with academic achievement across a wider range of paternal supportiveness [22]. Beyond direct associations, there is also evidence that positive parenting may buffer the effects of low socio-economic status on academic outcomes [23] and moderate the association between children’s early temperament and later self-regulatory capacities [24].

### 1.4. School factors, academic outcomes, and resilience

Extensive research efforts have sought to identify the key influences in school contexts that make a difference to children’s learning engagement and academic outcomes [25]. Geographic location is one factor linked with educational disadvantage from an early age, across many countries including in Australia and the United States [26,27]. Across Australia, population density is highest in the six state capitals. Despite policies and funding models that aim to support regional and remote education, children from urban schools tend to outperform students from rural and regional areas in national achievement tests [28].

Teachers’ qualifications, perceptions of their school environment, their self-efficacy, and relationship with students, all have the potential to drive variations in instructional practices and thus student outcomes [29]. Warm and responsive interactions between children and their teachers have important benefits for young learners and are considered a key mechanism that supports child engagement in learning activities [30]. This mechanism is also likely to influence teacher self-efficacy as a personal belief about their own effectiveness as a teacher. Hajovsky et al. [31], in a large longitudinal analysis of US data for 881 children, from 2^nd^ to 6^th^ grade, reported that teachers’ self-efficacy beliefs mediated the path between closeness of teacher-student relationships and mathematics achievement.

Parent engagement in school and children’s ‘school liking’ have also been identified as important considerations in determining academic outcomes. Parent engagement in school has a strong effect on children’s academic achievement [32] and is also involved in important mediated pathways. For example, in a longitudinal Australian study, the relation between school-based-parent involvement and children’s Grade 3 reading achievement was fully mediated by children’s classroom self-regulation behaviors as reported by teachers [33]. Children’s perspectives of the extent to which they like school are an important reflection of children’s positive school experiences, teacher responsiveness, and classroom engagement. Children’s school liking has been also linked with their ability to manage emotions and focus attention and academic competence [34].

### 1.5. The current study

There is much still to learn about the specific child, parenting, and school factors that may compensate for early learning vulnerability. While links have been established between children’s school readiness skills and subsequent learning outcomes, there has been less focus on distinguishing constructs that are robustly associated with academic resilience, given early learning vulnerability. While most studies of academic resilience have defined risk based on socio-economic disadvantage [7,35], we use an alternative approach, defining early risk based on teacher-identified, developmental vulnerability at school entry. Specifically, this study addresses:

**Research Question 1:** Are there children who display teacher-reported vulnerability at school entry who subsequently achieve better-than-expected academic outcomes in reading and numeracy by Grade 3 of school, who could be described as resilient?

**Research Question 2:** What child, parenting, and early school factors are associated with academic resilience by Grade 3, in comparison to children who remain vulnerable with continued low academic achievement though the school years?

The sample comprises child participants of a large longitudinal study of Australian children. This dataset has linked data from a national early development census completed at school entry (Preparatory year; age 5–6 years), along with extensive child, family, and school data collected when children were in Grade 1 (6–7 years). National assessment data for academic achievement collected when children were in Grade 3 (8–9 years) of school are also linked to this dataset. Therefore, the study investigated children’s academic resilience as identified in Grade 3 with reference to their competency at school entry (Preparatory).

## 2. Methods

### 2.1. Participants

This study uses data from *Growing Up in Australia*: *The Longitudinal Study of Australian Children* (LSAC) which is sponsored by the Australian Government Department of Social Services and comprises two cohorts of children, recruited to be representative of the Australian population. The focus for this research is the Baby (B) cohort of 5,107 children whose age at recruitment was 0 to 1 year, for whom data have been collected biennially since 2004. Sampling, recruitment, and study design details are provided elsewhere [36]. Briefly, children were selected through use of the national health insurance database (Medicare) using a two-stage clustered design. First, 311 postcodes were randomly selected, with children randomly selected within postcodes, stratified proportionate to postcode populations [36]. Due to postcodes covering relatively large geographic areas with many schools situated within each postcode, there is no clustering of children within schools or teachers in the dataset.

LSAC data collection every two years comprises parent and teacher questionnaires, computer-assisted interviews with parents and children, and direct assessments with the child. Several national administrative collections of data are linked with LSAC data, including three used in the current study (further detail in Measures): *Australian Early Development Census* (AEDC); *National Assessment Program–Literacy and Numeracy* (NAPLAN); and school characteristics (*MySchool)*. Specifically, the current study uses:

- AEDC linked data on children’s school readiness from 2009 (age 5–6 years; first year of full-time school, Preparatory [“Prep”]).
- LSAC data collected for the B cohort at Wave 4 in 2010 (age 6–7 years, Grade 1).
- *MySchool* linked data on characteristics of children’s schools (related to 2010).
- NAPLAN linked data on children’s school achievement (age 8–9 years; Grade 3).

The LSAC study is approved by the Human Research Ethics Committee of the Australian Institute of Family Studies, and all children, their carers, and others involved in the study provide informed written consent. Fully anonymized LSAC data is made publicly available for use by researchers with no further ethical approval required to conduct this secondary data analysis (as per the current study). The analytic sample for this study comprised those children for whom complete AEDC and LSAC data were available (*n* = 2,118, 41%). Differences between the selected sample and those not selected due to unavailability of linked data were tested (Table 1). The selected sample had a slightly higher socio-economic position than participants not selected, and were less likely to have Aboriginal or Torres Strait Islander status, or have a non-English, home language.

**Table 1:** Sample demographics of selected and excluded participants.

	Sample			
Socio-demographic characteristics	Included (*n* = 2,118)	Excluded (*n* = 2,989)	Significance	
	**n (*%*)**		**χ** ^**2**^	***p***
Female	1035 (49)	1462 (49)	.00	.50
Aboriginal and/or Torres Strait Islander	68 (3)	162 (5)	14.07	< .01
Home language other than English	171 (8)	381 (13)	28.08	< .00
	***M (SD)***		***F***	***p***
Socio-economic position (at age 6–7 years)	.00 (.73)	-.07 (.76)	10.72	< .01

### 2.2. Measures

In this section, we describe the measurement data from the *Australian Early Development Census* (AEDC) used to identify early vulnerability by teacher-report at school entry. Also described are the measurement data from the *Australian National Assessment Program for Literacy and Numeracy* (NAPLAN), used in these analyses, to differentiate children who show academic resilience at Grade 3 or continued academic vulnerability. A range of explanatory variables are also described, which are used in the analyses to identify child, family, school, factors associated with academic resilience.

Socio-demographic variables included as covariates in the current analyses were: child gender (0 = male; 1 = female); non-English speaking background (0 = English spoken at home; 1 = non-English language spoken at home); and Aboriginal or Torres Strait Islander status (0 = no; 1 = yes). The continuous variable included to describe family socio-economic position combined information on parental occupational prestige, parental education level, and household income, with higher scores reflecting a higher socio-economic position [37].

#### 2.2.1. Variables used to establish early vulnerability and later academic resiliency

*School entry developmental capabilities* were identified using data from the AEDC. Typically, across Australian states, children will be enrolled in full-time school at 5 years of age or have their 5^**th**^ birthday in the first six months of that school year (school years align with calendar years). Nationally, AEDC data are collected every three years by the Australian Government to monitor the school readiness of Australian children in their first year of full-time school. Teachers rate each child’s developmental capabilities based on their observations, on more than 100 items, across five developmental domains (physical health and well-being; social competence; emotional maturity; language and cognitive skills; communication skills and general knowledge).

The AEDC domain scores have established reliability and validity relative to other validated indices of child development [38]. In population-level reporting of AEDC findings by the Australian Government, domain scores are converted to percentiles, and children are assigned to one of four groups: children with the lowest 10% of scores are described as ‘developmentally vulnerable’; children with domain scores from the 10^th^ to 25^th^ percentile are described as ‘developmentally at-risk’. Other groups (25^th^ to 50^th^ percentile; and the top 50% of children) are considered as ‘developmentally on track’.

We maintain a specific focus on the AEDC language/cognitive domain to identify LSAC children whom we consider to be ‘academically vulnerable’ at school entry [3–5], to explore influences that contribute to their academic resilience at Grade 3. Prior studies [3–5] indicate that the AEDC domain score for language/cognitive skills is most predictive (among the five AEDC domains) of children’s later academic achievement in Grade 3 of elementary school (using NAPLAN data on reading comprehension and numeracy). Preliminary analysis (presented in S1 Appendix) confirmed this robust association between the AEDC language/cognitive domain at school entry and reading comprehension and numeracy achievement at Grade 3 for this cohort of students.

Though publicly available data on the AEDC categorizes only those children in the bottom 10% of scores as ‘developmentally vulnerable’, our analyses identified a gradient effect across all four of the percentile groups typically differentiated with AEDC data. Specifically, only those in the top 50% of scores in the language /cognition domain at school entry later performed at or above the grade level equivalent expectations specified for academic achievement in Grade 3 [39]. We therefore include in these analyses all children in the lower three AEDC percentile groups on language/cognition (i.e., all children at or below the bottom 50^th^ percentile, namely the AEDC groups designated lowest 10%, 10 - 25^th^ percentile, and 25^th^ - 50^th^ percentile) and describe these children as *Academically Vulnerable (n = 781; 36*.*87%)*. These children are the focus of our analytical models. Children in the top 50% of scores on the AEDC language/cognitive domain were considered *Strong in Prep* (n = 1,337; 63.13%), and are not included in our analytical models.

*Grade 3 academic achievement*. NAPLAN is an annual assessment of literacy and numeracy skills for all Australian school students enrolled in Grades 3, 5, 7, and 9. Children are assessed across five academic domains: Reading Comprehension, Writing, Spelling, Grammar and Punctuation, and Numeracy, with a score range in each domain of 0 to 1000. The tasks in NAPLAN tests are developed with reference to the nationally-agreed Statements of Learning that are considered essential for every child to achieve and progress successfully through school [39]. Grade level equivalent scores for each domain of achievement in Grade 3 [39] have been published by the Grattan Institute based on 2014 NAPLAN achievement data. In the current analyses, children’s scores for NAPLAN Reading Comprehension and Numeracy in Grade 3, assessed when children are typically aged 8–9 years, are used to establish children’s academic resilience.

#### 2.2.2 Variables used to explain academic resilience

A range of explanatory variables were included in these analyses using data from LSAC Wave 4 [40] and linked *MySchool* data, when children were aged 6–7 years, and enrolled in Grade 1 of school. Variables were derived from parent interview / questionnaire data and questionnaires completed by the child’s Grade 1 teacher. Direct assessments and interviews with children were also completed when the parent interview was conducted in the family home. Child characteristics, parenting, and school factors included in these analyses were those identified from previous studies as likely to have impacts on children’s academic achievement and/or were factors that protect against poorer educational outcomes for children at risk. These variables are described in Table 2. Bivariate correlations among these variables are presented in S2 Appendix.

**Table 2:** Variables used to explain academic resilience.

***Child factors***	
Emotional regulation	4 items (*Student Teacher Relationships Scale–Short Form* (STRS) [41]–e.g., ‘easily angry’, ‘remains angry’; & 1 item from *Strengths and Difficulties Questionnaire* (SDQ) [42] ‘has a temper’; used in prior LSAC studies [43–45]
Attentional regulation.	1 teacher-reported item—SDQ (‘has a good attention span’), & 5 items from *Social Skills Rating Scale* (SSRS) [46]–e.g., ‘pays attention’, ‘persists’, ‘is organized.’ As used in prior LSAC studies [43–45].
Sleep problems	1 parent-report item which asked whether, overall, the child had a sleep problem (no, mild, moderate, or severe problem). As used in prior studies [45,47].
Receptive vocabulary	Direct assessment using LSAC short form [48] of *Peabody Picture Vocabulary Test–Third Edition* (PPVT-III) [49].
Peer problems	5 teacher-report items of the peer problems subscale from the *Strengths and Difficulties Questionnaire* (SDQ) [42].
***Parenting factors* for mothers and fathers**	
Parental consistency	5 parent-report items (NLSCY) [50]; (10-point scale—1 = not at all to 10 = all of the time): e.g., ‘How often does this child get away with things that you feel should have been punished?’.
Parental warmth.	6 parent-report items from Child Rearing Questionnaire [51]; 5-point scale (never to almost always)–e.g., ‘How often do you express affection by hugging, kissing, and holding this child?’.
Parental anger	5 parent-report items adapted items from the NLSCY [50]; 5-point scale (never to almost always); e.g., ‘How often are you angry when you punish this child?’.
**School factors**	
Remoteness	LSAC remoteness area classification for family location: 0 = highly accessible to all services (e.g., urban area, major city) to 4 = very remote.
School sector	*MySchool* linked data (1 = government, 0 = non-government).
School size	*MySchool* linked data–total student enrolment.
Learning support teacher	1 teacher-report item (1 = yes and 0 = no).
Teacher perception of a positive work environment	6 teacher-reported items (NLSCY) [52]; (1 = strongly disagree to 5 = strongly agree); ‘e.g., I can rely on colleague’, ‘I am able to contribute to decision making’.
Teacher perception of school student behavior support	5 teacher-reported items (NLSCY) [52]); (1 = strongly disagree to 5 = strongly agree)–e.g., ‘There is consensus about how to discipline students’.
Teacher education level.	1 teacher-reported item (1 = high school completion to 7 = doctoral degree).
Teacher specialized training in early childhood	1 teacher-reported item (1 = yes and 0 = no).
Teacher self-efficacy	4 teacher-reported items (NLSCY) [52]; (1 = strongly disagree to 5 = strongly agree)–e.g., ‘I have a strong effect on academic achievement’, ‘I feel competent with managing behavioral and learning problems’.
Parent engagement in their child’s school	5 teacher-reported items (0 = no; 1 = yes)–e.g., ‘visited child’s class’, ‘talked to other school parents’, ‘attended school event’.
Teacher closeness to child.	5 teacher-reported items (*Student Teacher Relationships Scale–Short Form* (STRS) [41]; e.g., “I share an affectionate, warm relationship with this child.”
Child liking of teacher	1 child-reported item (3-point scale: 1 = no, 2 = sometimes, 3 = yes).
Child liking of school.	7 child-reported items [53]; (3-point scale: 1 = no, 2 = sometimes, 3 = yes)–e.g., ‘Is school fun?’, ‘Is school a good place to be?. Related to emotional aspects and academic aspects of school.

*Explanatory variables*, *representing child*, *parenting factors*, *and school factors*, *are drawn primarily from LSAC when the Baby Cohort children were 6–7 years old (Wave 4 data collection)*. *Full referencing details available from LSAC documentation or authors*.

### 2.3. Approach to analysis and missing data

**Fig 1:**
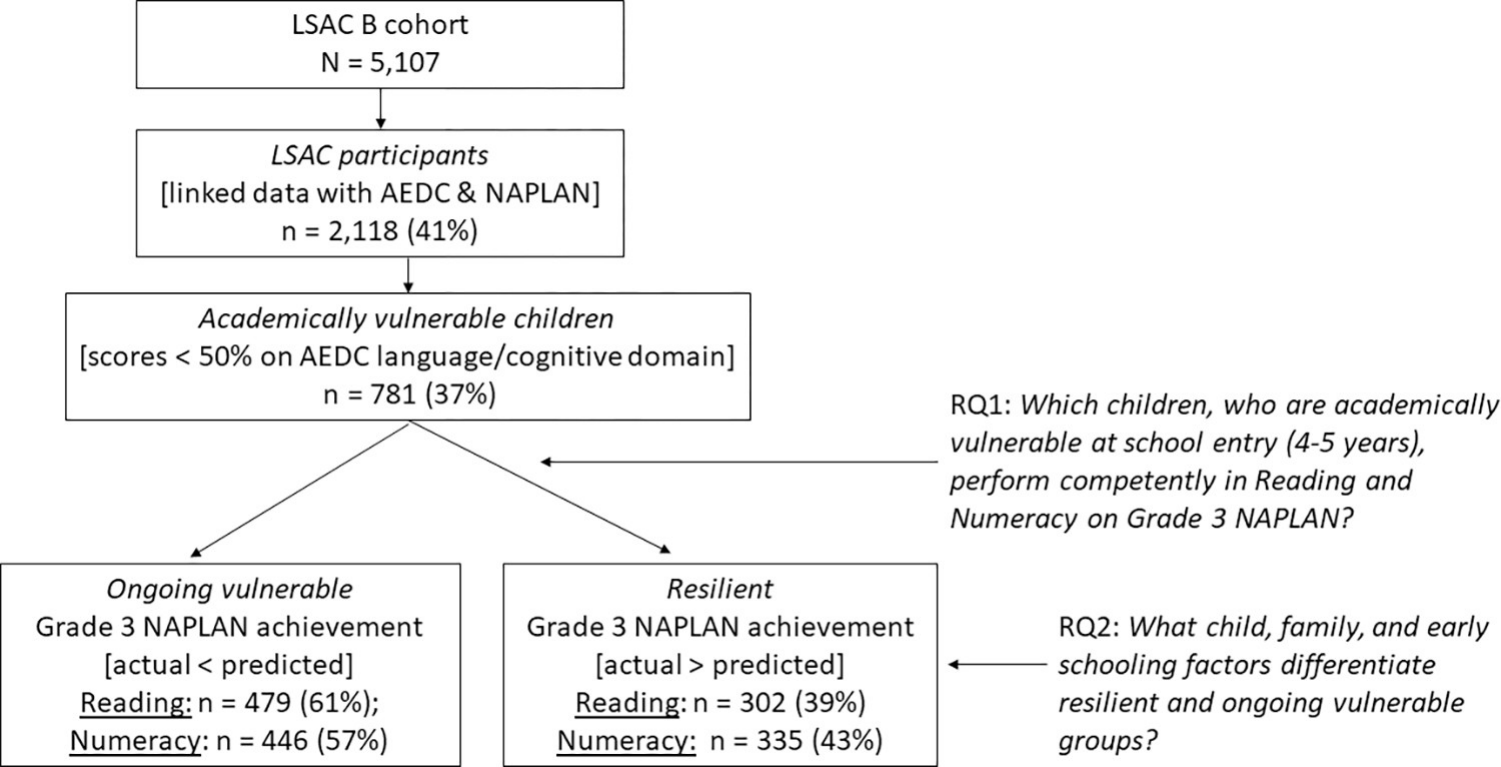
Participant sample and research questions at each stage of the study. LSAC B: Longitudinal Study of Australian Children Baby cohort; AEDC = Australian Early Development Census; NAPLAN = National Assessment Program Literacy and Numeracy.

Fig 1 depicts the participant sample and research questions at each analysis stage. As each subsequent stage of analysis built on the findings of the previous stage, the analytical approach at each stage is detailed within the Results section.

In relation to missing data, because of the selection process for the initial analytic sample (see 2.1 Participants), there were negligible missing data for the analysis pertaining to Research Question 1. Analysis for Research Question 2 focused on a sub-sample of our original sample, who were identified as academically vulnerable. Explanatory variables used in the modeling had missing data ranging from less than .01% (parent-reported data) to 24% (teacher-reported data). Data were considered missing at random (MAR) because it was unlikely that the presence of a missing value was related to the response that would have been provided [54]. We used multiple imputation in MPlus V8.2 to create 40 imputed datasets which were used for analysis. The imputation model included all the substantive and control variables described in Table 2 and included in the final model (as well as earlier measures for some of the substantive variables, drawn from Wave 2 of LSAC data when children were aged 2–3 years, namely: socio-economic position, parent-reported emotional and attentional regulation and sleep problem behaviors of children, and self-reported maternal and paternal hostility). The results presented are pooled across the 40 datasets (using TYPE = IMPUTATION in MPlus).

## 3. Results

### 3.1. Research Question 1: Identifying academically resilient children

These analyses identified a group of children who were assessed as academically vulnerable by their teachers at school entry (i.e., < 50% in distribution of scores on AEDC language/cognitive domain; n = 781). From within that group, children who then achieved better-than-expected outcomes on measures of academic achievement (i.e., NAPLAN reading and numeracy assessments by Grade 3 of school) were identified and delineated as being academically resilient at Grade 3.

To identify this academic resilient group at Grade 3, we used a calculation that compared *predicted* Grade 3 achievement scores to *actual* Grade 3 achievement scores. As detailed further in S3 Appendix, predicted scores for reading and numeracy on NAPLAN Grade 3 achievement tests were calculated using the formula: P_Achieve_ = M_Top50LG_−(*β* x SD), where P_Achieve_ was the predicted score on each relevant achievement test (reading or numeracy); *M*_Top50LG_ was the mean achievement score of the student group considered *not* vulnerable at school entry (i.e., *Strong in Prep*: those in the top 50% of scores in the language/cognition domain of the AEDC); *β* was the standardized regression coefficient (with respect to the dependent variable of reading or numeracy score) that explains the expected standard deviation difference in academic achievement scores for each of the three academically vulnerable groups (score ranges in the lowest 10%, 10–25%, and 25–50%) compared to ‘not vulnerable’ group (see Table C1 in S3 Appendix); and SD was the standard deviation of the mean of the overall sample scores for each achievement test. This meant that all children in the bottom 10^th^ percentile group on the AEDC were allocated the same predicted score, as were those children in the 10–25% group, and those children in the 25–50% group (Table C1 in S3 Appendix).

This was a fit-for-purpose formula that allowed the parameters of the initial regression models to be used to calculate predicted or expected scores of children who entered school academically vulnerable, with unique predicted scores produced for each of the three AEDC vulnerable groups. As the regression coefficient for each of the three strata within the academically vulnerable group differed (and differed for each of reading and numeracy), so too did the predicted score for each strata. This meant that the predicted score for children in the bottom 10^th^ percentile group at school entry (375.65 for reading; 349.98 for numeracy) was lower than the predicted score for those in the 10^th^ to 25^th^ percentile (405.03 for reading, 375.75 for numeracy), and for those in the 26^th^ to 50^th^ percentile (431.05 for reading, 404.30 for numeracy).

The difference between the predicted and actual scores for each NAPLAN achievement test was calculated for each child. Children with a positive difference (i.e., actual score equal to or higher than predicted score) were coded as *Resilient* (coded 1 for resilience variable). They met our definition of resilience by performing better than expected on Grade 3 NAPLAN achievement tests. Children with a negative difference score (i.e., actual score lower than their predicted score) were coded as *Ongoing vulnerable* (coded 0 for resilience variable). No child had achievement scores exactly as predicted. This allocation showed that 39% (n = 301) were resilient in reading comprehension at Grade 3 and 43% (n = 335) were resilient in numeracy. Looking across the reading and numeracy domains for each child, there were 54% (n = 421) children who were resilient for at least ONE of these two domains. Of these resilient children, 51% (n = 216) were resilient across both assessments, 20% (n = 86) were resilient only in reading, and 28% (n = 119) were resilient only in numeracy.

Socio-demographic differences among children identified as resilient compared to children who remained in the ongoing vulnerable group were explored using chi-square tests (for categorical variables) and ANOVAs (for continuous variables) and are shown in Table 3. Across both reading and numeracy assessments, children who were resilient were more likely to be non-Indigenous and had a higher socio-economic position, than children in the ongoing vulnerable group. Boys were more likely than girls to be resilient in numeracy.

**Table 3:** Socio-demographic characteristics of resilient children in each academic achievement domain.

	Grade 3 academic resilience	
	Reading	Numeracy
	n (%)	n (%)
Total resilient	302 (39%)	335 (43%)
Girls (n = 325; 42%)	120 (40%)	111* (33%)
Boys (n = 456; 58%)	182 (60%)	224* (67%)
Aboriginal and Torres Strait Islander (n = 40; 5%)	8* (3%)	8* (2%)
Non-Indigenous (n = 741; 95%)	294* (97%)	327* (98%)
Non-English speaking at home (n = 65; 8%)	26 (9%)	27 (8%)
English speaking at home (n = 716; 92%)	276 (91%)	308 (92%)
Mean socio-economic position:		
Resilient group	.02*	-.04*
Ongoing vulnerable group	-.33	-.31

* statistically significant difference between resilient and ongoing vulnerable groups at p < .05.

**Fig 2:**
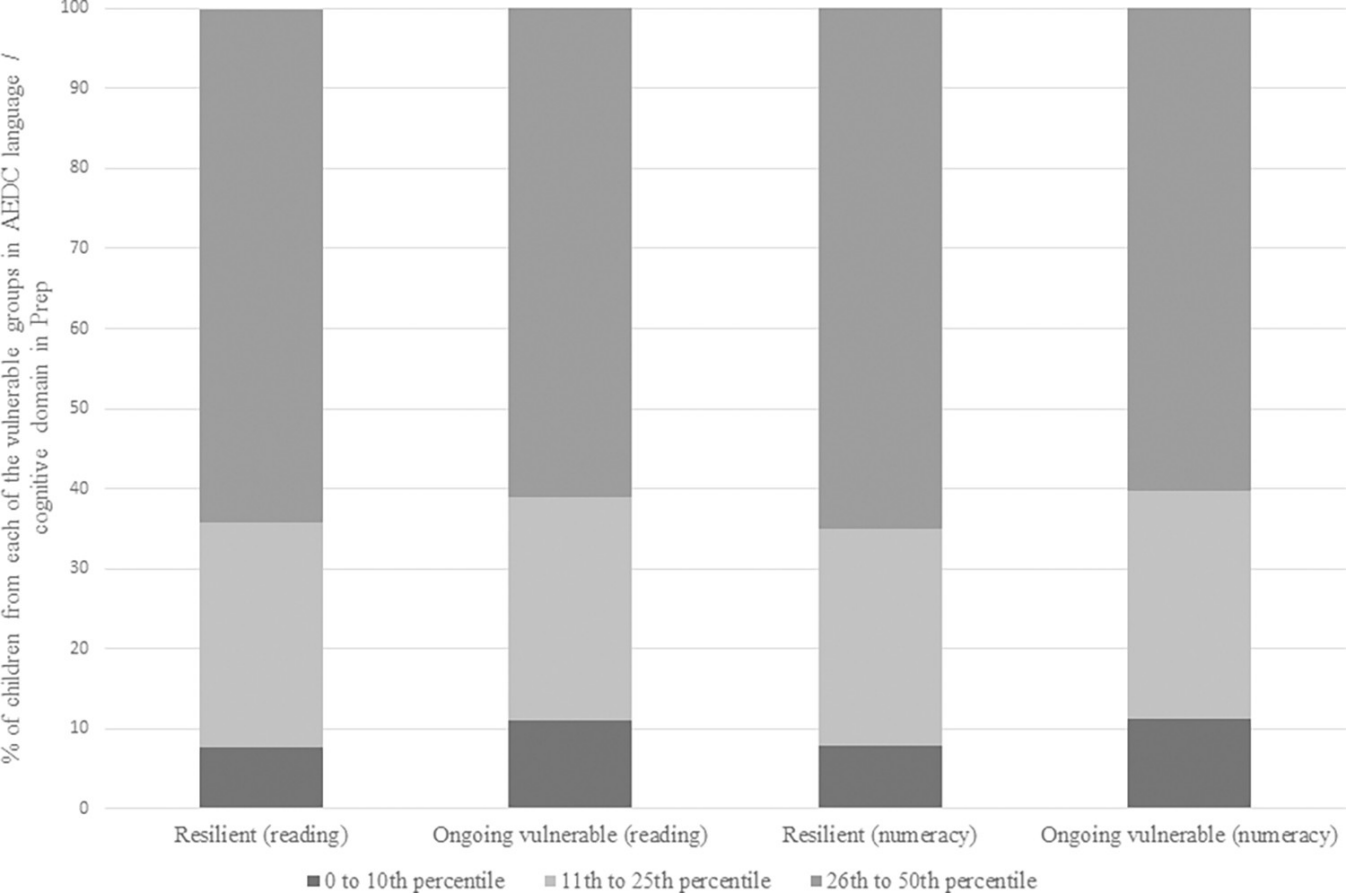
AEDC language/cognitive domain at school entry. Percentages of children from each of the vulnerable groups in Prep language/cognitive domain (AEDC), identified as resilient or ongoing vulnerable in each of reading and numeracy in Grade 3.

Fig 2 depicts the degree to which children in each of the three vulnerable percentile groups on the AEDC language/cognitive domain at school entry were represented in the resilient and ongoing vulnerable groups at Grade 3. Children in each percentile group were approximately equally likely to be identified as resilient or ongoing vulnerable for reading and numeracy in Grade 3. This demonstrates that it is not just those children in the 25^th^ to 50^th^ percentile group at school entry who have the capacity to close the gap between vulnerability at school entry and later school achievement but also children with greater vulnerability at school entry were also able to be classed as academic resilient at Grade 3 of school.

**Fig 3:**
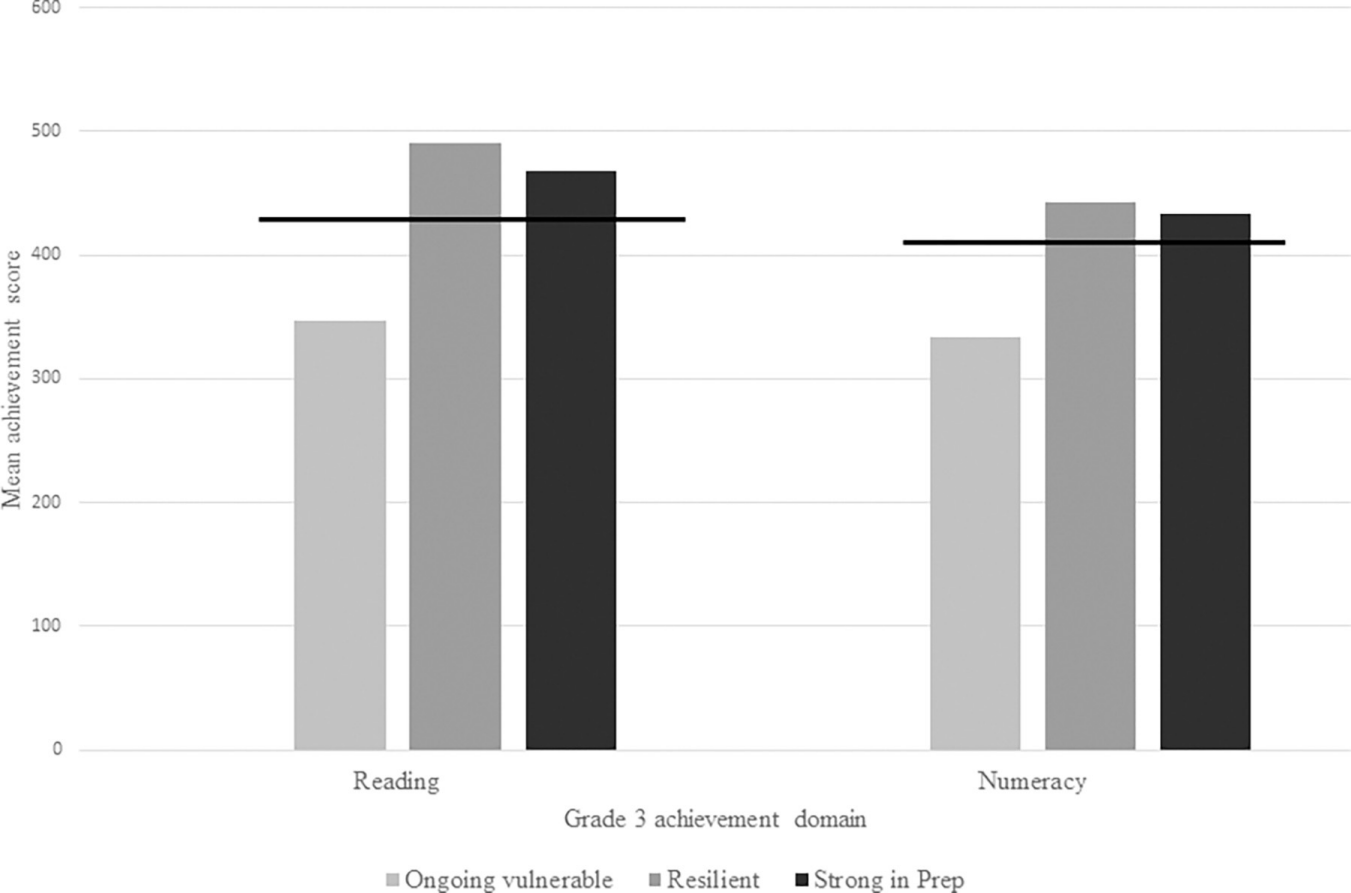
NAPLAN achievement data (Grade 3). Mean Grade 3 achievement scores in each domain for children identified as ongoing vulnerable, resilient, and strong in Prep. Horizontal black lines show Grade 3 level equivalent scores published by the Grattan institute based on 2014 NAPLAN achievement data [39], for the reading and numeracy domains. These show that both resilient and strong in Prep children performed above Grade 3 equivalent level.

We then compared Grade 3 achievement scores of students across three groups–ongoing vulnerable, resilient, and strong at Prep (recall strong at Prep children had an AEDC language /cognition score above the 50^th^ percentile). At the mean group level, children in the resilient group, due to their better-than-expected academic scores in Grade 3 at the individual level, performed as well (or better, in the case of reading comprehension) than children in the strong at Prep group. Importantly, although children only had to perform ‘better-than-expected’ according to the score calculations, in fact, resilient children on average performed above published, grade level equivalent scores [39] for reading and numeracy, shown by the black horizontal lines in Fig 3. As expected, children in both the resilient and strong at Prep groups had significantly higher achievement than children in the ongoing vulnerable group but did not differ in achievement levels from each other (tested with ANOVA, applying Bonferroni corrections).

### 3.2. Research Question 2: What factors explain the closing the gap pathway for academically resilient children?

These analyses explored associations between child, parenting, and school factors and children’s academic resilience in Grade 3, focusing on the group of children identified as academically vulnerable (n = 781; scores < 50% on AEDC language/cognition domain) who, subsequently, were classified as either resilient or ongoing vulnerable by predictive scores for NAPLAN achievement tests at Grade 3. Table 4 presents the group means on a range of explanatory variables tested, which identified significant differences between groups using ANOVAs (resilient children compared to ongoing vulnerable children); also see Table D1 in S4 Appendix for all bivariate comparisons conducted.

**Table 4:** Significant between-group differences on mean scores between resilient and ongoing vulnerable groups on explanatory variables.

	Reading		Numeracy	
	Resilient	Ongoing vulnerable	Resilient	Ongoing vulnerable
*Child factors*				
Attentional regulation	18.96**	16.71	18.89**	16.62
Sleep problems	1.36	1.37	1.26**	1.44
Vocabulary	75.21**	72.28	74.61**	72.52
Peer problems	5.96**	6.32	6.00*	6.31
*Family factors*				
Maternal consistency	4.33*	4.21	4.34**	4.20
Paternal consistency	4.27**	4.09	4.29**	4.06
Maternal anger	1.71**	1.84	1.70**	1.87
Paternal anger	1.71	1.78	1.68**	1.82
*School factors*				
Remoteness^	.73**	.93	.73**	.94
% Government school	68.4	69.1	64.8*	71.9
Teacher self-efficacy	4.46	4.42	4.49*	4.40
Parent engagement	3.33*	3.12	3.44**	3.04

^ higher scores indicate a more remote area with the anchor of the scale (zero) indicating urban locations with higher service access; # total enrolment

** significantly different to the ongoing vulnerable group at p < .01

* significantly different at p < .05.

Across areas of assessment, children who were identified as academically resilient at age 8–9 years (Grade 3), when compared to ongoing vulnerable peers, had higher teacher-rated attentional regulation, higher scores on the direct test of receptive vocabulary, fewer teacher-reported peer problems, lower levels of mother-reported parenting anger, higher levels of mother- and father-reported consistency, lived in urban areas (e.g., major cities or inner-regional areas with good access to services), and had higher rates of parent school engagement reported by teachers, when children were aged 6–7 years. For numeracy achievement only, children who were resilient also had fewer parent-reported sleep problems, lower levels of father-reported anger, had teachers with higher levels of self-efficacy at age 6–7 years, and were more likely to attend a non-government school compared to a government school. There were no mean level differences between the resilient and ongoing vulnerable children in children’s teacher-reported emotional regulation, mother- and father-reported parenting warmth, level of access to learning support in the classroom, teacher-reported positive work environment, whole-school behavior approach, teacher level of education or being trained in early childhood, parent-reported engagement in school, teacher-reported closeness to child, extent to which children reported liking their teacher or their teacher being nice to them, child report of liking the social/emotional aspects or academic aspects of school, or school size (estimates detailed in Table D1 in S4 Appendix).

**Fig 4:**
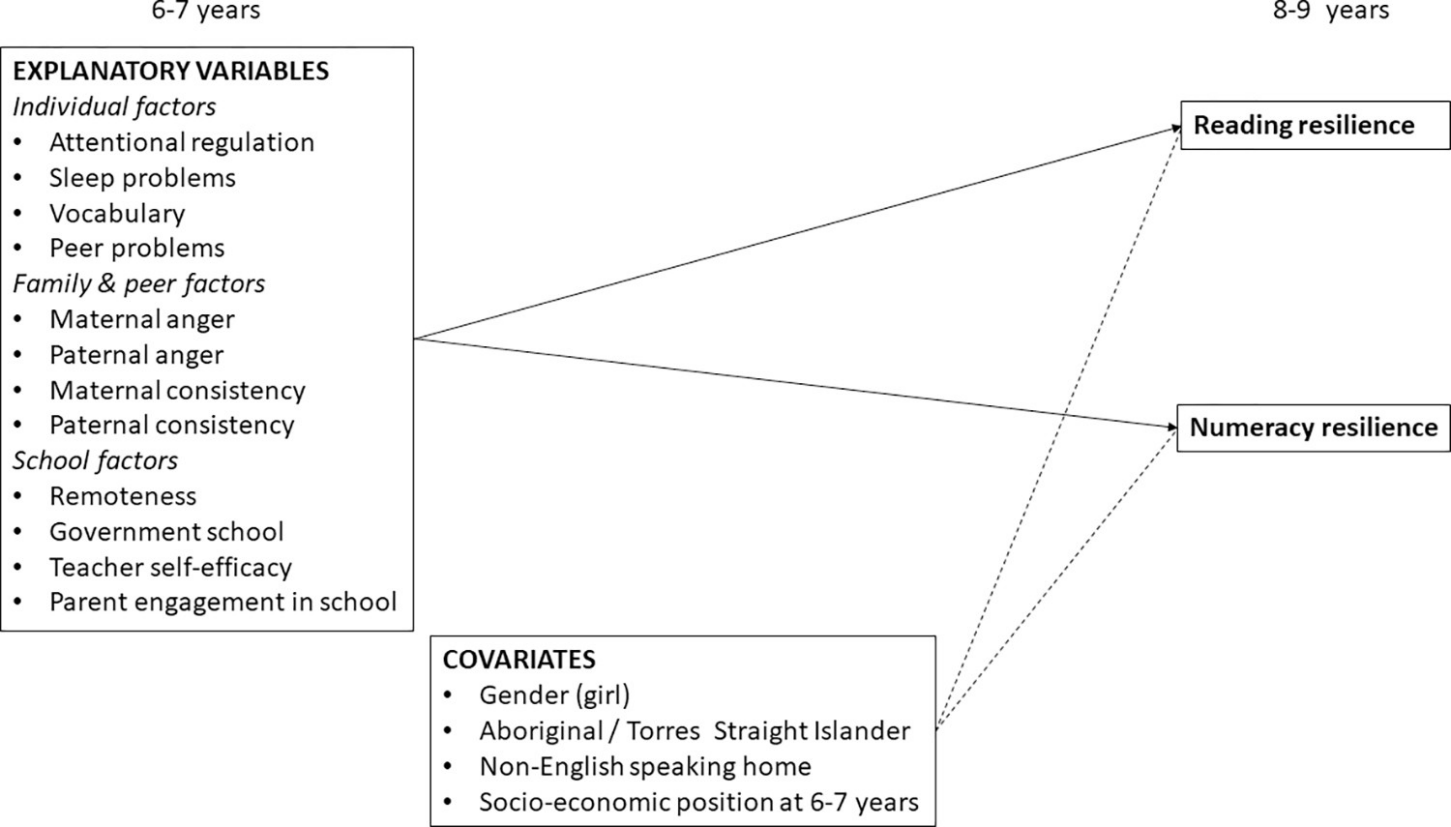
Final model examining association between explanatory variables at age 6–7 years and membership of academic resilience groups in each achievement domain at Grade 3 (age 8–9 years).

A single path model was developed to determine the relative strengths of association of explanatory variables (at age 6–7 years) in relation to membership of the academically resilient group for both domains of achievement (age 8–9 years). As the outcome measures were binary (resiliency group membership: 0 = no, 1 = yes), the path estimates are equivalent to a multinomial logistic regression. Explanatory variables included in the path model were those that showed significant bivariate relations (in the initial chi-square and ANOVA analyses) with resilient group membership for either reading comprehension or numeracy. Lack of multicollinearity among included variables was confirmed, with all Variance Inflation Factors < 2. First, a fully saturated model was estimated in which all potential paths from each explanatory variable to resilient group membership in the respective academic domain were estimated. In a second step, socio-demographic covariates were added in relation to the outcomes of academic resiliency in each domain (Fig 4).

Table 5 presents the standardized estimates for the fully adjusted model that included all covariates. These show that children’s teacher-reported attentional regulation and assessed receptive vocabulary at age 6–7 years were significantly associated with being in the academic resilient group for reading and numeracy by Grade 3. Higher levels of paternal consistency were associated with membership of the reading resilient group. Fewer sleep problems and attending an urban school in a major city or inner regional location was significantly associated with numeracy resilience. In relation to the covariates, boys and children from higher socio-economic families were more likely to be resilient. The model accounted for 32% of variance in prediction of both reading and numeracy resilient group membership.

**Table 5:** Standardized estimates from the path model examining explanatory variables, covariates, and academic resilience group membership.

Area of resilience	Reading	Numeracy
*Individual factors*		
Attentional regulation	.31**	.35**
Sleep problems	.04	-.10*
Vocabulary	.29**	.19**
Peer problems	-.00	.10
*Family and peer factors*		
Maternal anger	-.08	-.08
Paternal anger	.01	-.07
Maternal consistency	-.05	-.05
Paternal consistency	.13*	.06
*School factors*		
Remoteness^	-.07~	-.11*
Government school	.06	-.05
Teacher self-efficacy	.01	.08
Parent engagement	-.07	.08
*Covariates*		
Girls	-.19*	-.65**
Aboriginal / Torres Strait	-.23	-.27
Non-English speaking	.16	.03
Socio-economic position	.23**	.21**

** statistically significant association at p < .01

* statistically significant at p < .05; ~ statistically significant (at p < .05) in the unadjusted model but not in the fully adjusted model that included all covariates

^ higher scores indicate a more remote area.

## 4. Discussion

Across national contexts, significant differences in developmental competencies exist between children when they begin school [27]. Such differences are often associated with family and community socio-demographic factors, as a consequence of structural inequities in access to resources within societies and the impact of stress on parents and children. While acknowledging systemic inequity, it remains important to consider how early learning gaps present at school entry can be addressed in classroom contexts. The experience of school for many students not only fails to close the learning gaps [4], but often widens them, with these gaps maintained across the school years [39]. This study identified an important group of academically resilient children who entered school academically vulnerable yet closed the learning gap by Grade 3. For these students, their receptive vocabulary skills and attentional regulation skill development, as measured in these analyses, during the early school years set them apart from those that remained on a low attainment trajectory. It is possible that when early classroom experiences focus on specific cognitive and non-cognitive skill development, as appropriate to the needs of individual children, there may be more opportunities for children to follow a resilient pathway.

### 4.1. Identification of academically resilient children

In this study, approximately two in five of the children identified as vulnerable at school entry showed academic resilience (39% resilient in reading comprehension; 43% in numeracy). Importantly, children in the academic resilient group at Grade 3, performed, on average, at least as well as children who entered school with stronger skills in the language/cognitive domain (i.e., performing above grade level equivalent scores at Grade 3). This included children with the largest learning gaps to close, who entered school with scores in the bottom 10% of the language/cognitive AEDC scores and who were as likely as those just under the 50^th^ percentile to perform better than expected by Grade 3. Thus, closing the gap in academic competencies does appear possible, regardless of the magnitude of the gap.

Key socio-demographic differences between the resilient and ongoing vulnerable groups also provided evidence about which children are more, or less, likely to display academic resilience. Non-Indigenous children were more likely to show academic resilience. However, once family socio-economic position was included in the final model, this significant effect was no longer evident. This finding suggests that it was not cultural status, per se, but associated socio-economic differences that contributed to resilient pathways. Children from higher socio-economic families, although identified as academically vulnerable at school entry, were more likely to show academic resilience. Children from non-English speaking homes were equally as likely as those from English-speaking homes to be in the academically vulnerable group at school entry (Table 3), and equally likely to show a resilient pathway. This finding suggests that coming from a non-English speaking background and entering school with poorer language/ cognitive skills (at least as demonstrated in English) does not preclude these children from closing the competency gap over time. Given that 25% of children in Australia enter school with a language background other than English [55], this is an important finding. It is possible that this finding reflects educational investments in their children by some immigrant families. In other analyses with LSAC data [56], it was reported that at school entry, non-Anglo immigrant children spend more time on educational activities that non-immigrant Australian children. It is also acknowledged that elements of systemic inequity not measured or analyzed in this study are likely to play an important role in determining which children show resilience over time and which do not. For example, it is documented that experiences of racism by Indigenous children in Australia, and their experience of learning environments and educational policy that were designed for students from a Western background only, impact on academic achievement for Aboriginal and Torres Strait Islander children [57,58].

The finding of this study on gender differences in resilience group membership, favoring boys who were more likely to show resilience in numeracy than girls, is difficult to interpret. Understanding gender effects on children’s mathematics performance requires an in depth consideration of sociocultural influences [59] present from early childhood. Nonetheless, the findings here suggest that efforts to address mathematics participation, confidence, and achievement by girls that have become common in later elementary and secondary school, might be best implemented from school entry.

### 4.2. Factors that support academic resilience

Children’s attentional regulation and receptive vocabulary skills (assessed age 6–7 years) emerged as key factors in multivariate models that distinguished children classified as academically resilient at Grade 3 (aged 8–9 years), compared to their ongoing vulnerable peers. This finding confirms prior studies that identify self-regulation as key to learning [13] and as a moderator of early risk to later achievement relations [17]. Children who can pay attention, resist distraction, persist in completing tasks, and organize their learning materials in the busy classroom environment will have more opportunity to capitalize on the learning experiences provided. Vocabulary, and overall oral language competence, are known to support reading comprehension development [60]; thus, it is no surprise that children with stronger vocabulary skills, despite early vulnerabilities, have higher academic achievement over time.

Paternal consistency was identified as a key contributor to academic resilience, in relation to reading achievement specifically, over and above the role of other variables in the model, including maternal consistency. Other studies have documented unique contributions of fathers in early childhood, controlling for maternal influences, including links between paternal warmth and children’s self-regulation and prosocial skills [61], and between fathers’ engagement in shared book-reading and children’s vocabulary development [62]. It has been suggested that indices of paternal engagement in models that involve both maternal and paternal factors, and children’s development, may act as a proxy for an overall richer home learning and family environment [62]. It is possible that in the current study paternal consistency indexed a more structured and supporting home learning environment overall, and perhaps high levels of co-parenting agreement and support.

Resilient children, compared to those who remained vulnerable, also showed fewer peer problems, fewer behavioral sleep problems, higher levels of parenting consistency by mothers, lower levels of parenting anger by mothers and fathers, higher levels of parental engagement in children’s school, and higher levels of teacher self-efficacy in bivariate analyses (ANOVAs), though these effects attenuated in the final multivariable model. These findings extend prior work identifying children’s sleep [15] and social competence [14], along with positive and consistent parenting [20,21], parental engagement in school [33], and teacher self-efficacy [31] as correlates of achievement in children, demonstrating that they also differentiate resilient children from their peers who remain vulnerable during the early school years.

It is likely that a mix of mediating and moderating pathways within children’s bioecological worlds, that include self-regulation, vocabulary, sleep health, parenting, and teacher characteristics, support academic resilient pathways. For example, positive parenting is known to support children’s self-regulation development [63] which in turn supports language learning [64] and academic achievement [13]. Language skills also predict growth in self-regulation over time [65]. Healthy sleep in children is known to support self-regulation development [63] as well as being directly predictive of achievement [16]. Future studies should aim to explicate these complex interactions across the transition to school and early school years period, which would help to identify the most salient modifiable factors upon which to intervene and at what time.

### 4.3. Implications for policy and practice

Our findings suggest that achieving an academically resilient pathway across the early school years is possible and, in this sample, was not uncommon. This is encouraging and points to the need for substantial ongoing investment and focus on the early years of elementary school. In many countries, substantial policy and public investment initiatives in recent decades have targeted the preschool years [66] due in part to: a) increasing evidence of the predictive nature of school entry competencies for longer term outcomes [4]; b) increasing knowledge that achievement gaps are most easily addressed early before they become entrenched; and c) an economic argument for the cost benefit of investing early to prevent later economic and societal costs of sustained underachievement [67]. While the value of investment in earlier intervention and prevention prior to school entry cannot be denied, our findings suggest that ongoing investment and effort in the early years of school is an additional worthwhile pursuit. The transition to school is a key milestone in family life and thus has high potential for intervention and remediation. Further, in most developed countries, the experience of school is a near-universal experience for children, meaning that high quality early classrooms that cater well for individual children, present a powerful population-based approach to address early developmental vulnerabilities.

Findings here and elsewhere suggest that teacher-report of children’s school entry competencies appropriately identifies students at risk of poor achievement three years later [3–5]. This is important given that substantial investment of time and money has been made in the AEDC and similar indexes internationally. It is important to note that, while standard AEDC reporting practices classify children in the lowest 10% of domain scores as developmentally vulnerable and children in the 11^th^ to 25^th^ percentile group as ‘at-risk’ [55], results presented here suggest that vulnerability to poorer longer term academic achievement is not restricted to these groups. A clear gradient effect was demonstrated and crucially, only children who were in the top 50% of scores in the AEDC language/cognitive domain performed on average, at or above the specified grade level equivalent score for Grade 3 [39]. This suggests that early school efforts to address developmental competencies on school entry should not be restricted to those in the lowest bands of AEDC scores, where the need for support might be more immediately obvious. Further, while this and other studies point to the predictive utility of the language/cognitive domain in terms of later achievement scores, vulnerability in any one school entry domain (including social and emotional competencies and physical health) has been shown to increase the risk of scoring poorly in standardized national measures until Grade 7 [5,68]. This suggests that a multi-dimensional approach to understanding school readiness that includes measures beyond cognitive and language capacities should be maintained.

Children in this research, who had ‘below average’ language/cognitive skills at school entry, along with poorer vocabulary and attentional regulation skills in the early years of school, over and above all other bioecological variables included, had the poorest outcomes. This suggests that when early educational experiences help to build these skills (and others that support academic resilience not measured in this study), it may be possible to stimulate resilient pathways for more children. In fact, given our variables used to explain resilience were collected in the final quarter of Grade 1 (second year of formal schooling), it is likely that for many children in our academically resilient group, the experience of school had already exerted a positive and important impact on these key skills. Importantly, approaches to supporting self-regulation and development of vocabulary can benefit all students, so a greater focus on these capabilities by teachers in the early years of school is crucial. For example, a focus on self-regulatory capacity building, in place of punitive behavioral management approaches in classrooms is required [69]. Interventions that focus on components of self-regulation (e.g., the executive functions) have been successful in narrowing the achievement gap in low achieving students [70], and can have multiple benefits including enhancing peer relationships [71].

Further, school-based approaches to supporting positive parenting, and supporting families to address children’s sleep problems and addressing parent engagement in children’s education can also enhance resilient pathways for children. Evidence for schools as sites for parent education [72] and family sleep education [73] has been documented as feasible and acceptable, yet relatively few schools deliver evidence-based parenting programs [74]. A recent meta-analysis of family-school partnership interventions found evidence for positive impact for children’s academic achievement, as well as social-emotional-behavioral outcomes [75]. Schools have an important and systematic role to play in supporting academic resilience in children, particularly for those that present with early risk factors. However, beyond the extent to which schools build academic skills in children, considered their core business, there appears untapped potential to provide a broader platform through which other important resilience factors could be boosted to improve children’s learning outcomes.

### 4.4. Limitations and future directions

Although this study has several strengths, including a longitudinal design, large sample, multiple and independent measures spanning teacher-report, parent-report, and direct assessments, and the use of linked administrative datasets, it is not without limitations. Given sample selection procedures based on available data, the sample was no longer nationally representative. Measures of school, classroom, teacher, peer, and child level factors were broad and brief, limiting the extent to which a nuanced understanding of the role of these factors in academic resilience could be explored.

Future studies should address these limitations as well some of the gaps not addressed by the current study. Students who entered school with above mean scores in the language/cognitive domain (developmentally strong at school entry) were not studied further. While it could be expected that most of these students performed at or above grade level expectations at Grade 3, there will likely be a subgroup of under-performing students whose experiences warrant further examination. Additionally, our analyses did not distinguish between the three groups whose school entry language/cognitive skills (as measured by the AEDC) ranked below the 50^th^ percentile in terms of whether equivalent degrees of academic resilience were observable within each group. Further, while the focus here was on academic resilience, social-emotional resilience is equally important for long term health and wellbeing [13], and so future studies should investigate these non-academic outcomes. While the language/cognitive domain of the school entry measurewas the strongest predictor of academic achievement in Grade 3 [3–5], it is likely that other domains of the school entry measure (AEDC), particularly the social and emotional domains, would be more highly predictive of other important outcomes of interest such as mental or physical health or social-emotional functioning. Future research should seek to produce evidence for this hypothesis and identify social-emotional resilient pathways and factors that support these. Longitudinal follow-up of children in the LSAC sample will also enable future studies to examine whether the trajectory of academic resilience is maintained (or not) over time using reading and numeracy assessments collected in Grades 7 and 9, and also compare how the ongoing vulnerable groups fare in comparison to their academically resilient peers.

## 5. Conclusion

Achievement gaps associated with socio-demographic gradients appear to be widening across many national contexts, but relatively little is known at a population level about the groups of vulnerable students at school entry who are able to close the academic competency gap across the school years, or the factors which enable them to achieve better-than-expected outcomes. This study used a large longitudinal dataset in an innovative way to identify developmentally vulnerable children at school entry and factors that contributed to an academic resilient pathway by Grade 3 for a subset of these children. Children may present to school with developmental vulnerability for a range of reasons, including limited access to enriching experiences in quality early education and care settings, or quality home learning environments. The findings of this study suggest that if key resilience factors of attentional regulation and vocabulary skills are boosted early, then children will be better positioned to take advantage of the learning opportunities available when they begin school. The corollary is that for children who face persistent challenges in attentional regulation and vocabulary development across the early school years, the findings here suggest that considerable learning potential and opportunities to be successful through education are lost. Schools should be part of systematic approaches to addressing a range of factors in the environments that children experience to promote the best possible educational outcomes. Education policy and practice can use this research evidence to support informed decisions about the focus and allocation of resources that can improve educational outcomes for all students, particularly those who may be most vulnerable when they begin school.

## Supporting information

S1 Appendix Preliminary analysis: Predicting Grade 3 achievement from school entry competency and identifying vulnerable children. (DOCX)

S2 Appendix Bivariate correlations among explanatory variables. (DOCX)

S3 Appendix Formula for the calculation of predicted scores, demarcating academically resilient verses ongoing vulnerable children. (DOCX)

S4 Appendix Descriptive differences on mean scores between academically resilient and ongoing vulnerable groups on explanatory variables. (DOCX)
