# Supplementary material for: Academic resilience from school entry to third grade: Child, parenting, and school factors associated with closing competency gaps
Source: PLoS One. 2022 Nov 30;17(11):e0277551. doi: 10.1371/journal.pone.0277551 (PMC9710847; doi:10.1371/journal.pone.0277551)
Supplement: S1 Appendix — (DOCX) [file pone.0277551.s001.docx]

**Appendix A**

# Paper: *Academic resilience from school entry to Grade 3: Child, parenting, and school factors associated with closing competency gaps.*

## **Preliminary analysis: Predicting Grade 3 achievement from school entry competency and identifying academically vulnerable children**

This supplementary material presents preliminary analyses conducted to confirm the predictive validity of the Australian Early Development Census (AEDC) domains at school entry in relation to Grade 3 academic achievement.

The research questions were:

1. *What is the relative strength of associations between school entry competencies and Grade 3 achievement?*
2. *Which school entry developmental domain is the strongest predictor of later Grade 3 achievement and which children can be considered vulnerable to poorer academic achievement given their school entry developmental competency scores?*

Using a variable-centred approach, two regression analyses in Mplus Version 7.11 (Muthén & Muthén, 1998 – 2012) were run, one for each of the achievement outcomes of reading comprehension and numeracy. The predictor variables were percentile category membership on each of the five AEDC domains (bottom 10%; 10^th^ to 25^th^ percentile; 25^th^ to 50^th^ percentile; and top 50%) with estimates shown in Table A1.

The school entry developmental domains of *communication and general knowledge* and *language and cognitive skills* were both consistently predictive of academic achievement across all domains, with language / cognition having the stronger associations.

While publicly available data on the AEDC categorises only those children in the bottom 10% as *developmentally vulnerable* at school entry, the data presented below in Table A1 suggest that there is a gradient effect across all percentile groups with only those in the top 50% of scores in the language /cognition domain at school entry performing at or above grade level equivalent expectations for academic achievement in Grade 3 (see Figure A1; Grattan). We therefore considered all children in these groups (bottom 10%, 10 to 25^th^ percentile, and 25^th^ to 50^th^ percentile) **academically vulnerable.** Students in the top 50% of scores on the AEDC language / cognitive domain were considered **strong in Prep** and are used as a descriptive comparison across the study, but not analysed in detail.

**Table A1** *Standardized regression estimates for each of the lower percentile groups in developmental competency at school entry across five domains, compared to those in the top 50% of developmental competency in each domain. These represent change in Grade 3 academic achievement scores in standard deviation units for each of the lower percentile groups, compared to the top 50% group.*

| AEDC domain | Percentile group in competency level | Reading achievement Grade 3 | Numeracy achievement Grade 3 |
| --- | --- | --- | --- |
| Physical | Lowest 10% | .08 | -.02 |
|  | 11^th^ – 25^th^ percentile | -.01 | -.07 |
|  | 26^th^ – 50^th^ percentile | -.03 | -.08 |
| Social | Lowest 10% | .01 | -.13 |
|  | 11^th^ – 25^th^ percentile | -.05 | -.11 |
|  | 26^th^ – 50^th^ percentile | -.08 | -.15** |
| Emotional | Lowest 10% | .05 | .26* |
|  | 11^th^ – 25^th^ percentile | .10 | .12 |
|  | 26^th^ – 50^th^ percentile | .07 | .08 |
| Language / cognition | Lowest 10% | -1.10** | -1.20** |
|  | 11^th^ – 25^th^ percentile | -.75** | -.83** |
|  | 26^th^ – 50^th^ percentile | -.44** | -.42** |
| Communication / general knowledge | Lowest 10% | -.63** | -.36** |
|  | 11^th^ – 25^th^ percentile | -.35** | -.19* |
|  | 26^th^ – 50^th^ percentile | -.26** | -.14** |

The regression estimates shown in Table A1 above indicate that percentile group membership in the language / cognition domain of AEDC in Prep is the most strong and consistent predictor of Grade 3 reading and numeracy achievement. Examining Grade 3 achievement scores for each of the four percentile groups of the AEDC language / cognition domain shows a gradient effect (Figure A1). That is, children in the top 50% of language / cognition scores in Prep (**strong in Prep** group) achieve on average significantly higher scores in all NAPLAN domains in Grade 3, with the mean score of children in each of the lower percentile groups substantially lower. Given year level equivalent scores published by the Grattan Institute (Goss, Sonnemann, Chisholsm, & Nelson, 2016) based on 2014 NAPLAN data for reading and numeracy (shown with black horizontal lines in Figure A1), it appears that it is ONLY children in the top 50% of scores (strong in Prep) that achieve on average at or above the year level equivalent score for Grade 3.


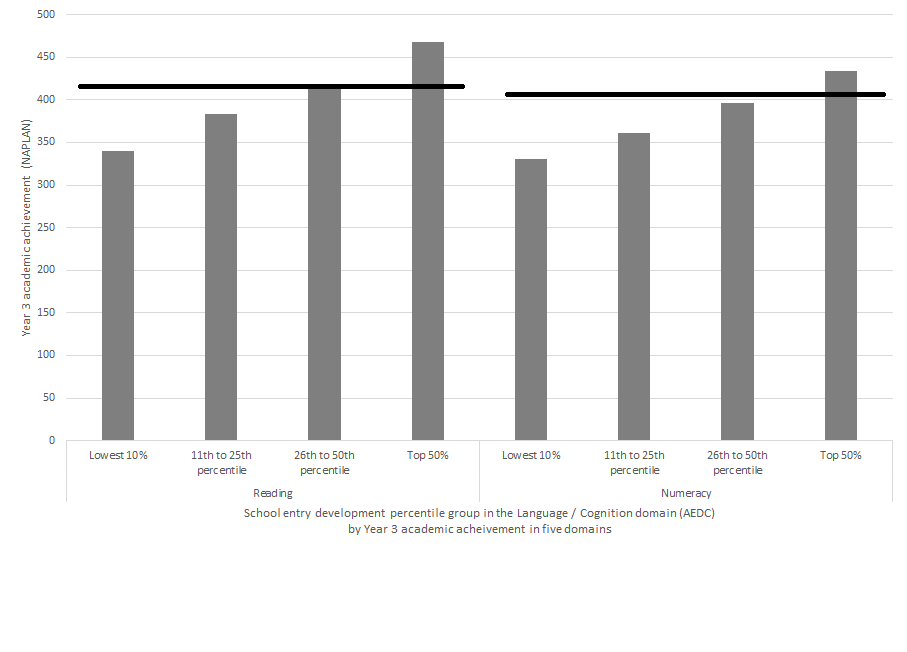


**Figure A1** *Mean academic achievement scores on third grade reading comprehension and numeracy domains for students in each percentile group of school entry developmental status on the language / cognition domain. Horizontal black lines show Grade 3 level equivalent scores published by the Grattan institute based on 2014 NAPLAN data (Goss et al., 2016) for the reading and numeracy domains. These indicate that only children in the top 50% of scores in the AEDC domain of language/cognition achieved above Grade 3 equivalent level.*

Grouping of children into strong in Prep and academically vulnerable groups based on the above rationale resulted in 63% of children in the strong in Prep group, and 37% in the academically vulnerable group. Analyses of the socio-demographic differences between the groups (Table A2) found that boys and Aboriginal and Torres Strait Islander children were more likely to be in the developmentally vulnerable group. This group also had a significantly lower family socio-economic position. Children with a home language other than English were equally as likely to be in the strong in Prep or developmentally vulnerable group.

**Table A2** *Socio-demographic differences between academically vulnerable and strong in Prep groups*

|  | **Group** | |  | |
| --- | --- | --- | --- | --- |
| **Socio-demographic characteristics** | **Strong in Prep**  **(*n* = 1,337, 63%)** | **Academically vulnerable**  **(*n* = 781, 37%)** | **Statistical Test** | |
|  | **n (*%*)** | | **χ^2^** | ***p*** |
| Female | 710 (53) | 325 (42) | 26.05 | <.01 |
| Aboriginal and/or Torres Strait Islander | 28 (2) | 40 (5) | 14.51 | <.01 |
| Home language other than English | 106 (8) | 65 (8) | 28.08 | <.40 |
|  | ***M (SD)*** | | ***F*** | ***p*** |
| Socio economic position (at 6-7 years) | .11 (.73) | -.19 (.73) | 90.81 | <.01 |
