# Supplementary material for: Academic resilience from school entry to third grade: Child, parenting, and school factors associated with closing competency gaps
Source: PLoS One. 2022 Nov 30;17(11):e0277551. doi: 10.1371/journal.pone.0277551 (PMC9710847; doi:10.1371/journal.pone.0277551)
Supplement: S2 Appendix — (DOCX) [file pone.0277551.s002.docx]

|  |  | **Appendix B. Bivariate correlations among explanatory variables** | | | | | | | | | | | | | | | | | | | | | | | | |  |
| --- | --- | --- | --- | --- | --- | --- | --- | --- | --- | --- | --- | --- | --- | --- | --- | --- | --- | --- | --- | --- | --- | --- | --- | --- | --- | --- | --- |
|  |  |  | 1 | 2 | 3 | 4 | 5 | 6 | 7 | 8 | 9 | 10 | 11 | 12 | 13 | 14 | 15 | 16 | 17 | 18 | 19 | 20 | 21 | 23 | 24 | 25 | 26 |
| 1 |  | Emotional regulation | 1 |  |  |  |  |  |  |  |  |  |  |  |  |  |  |  |  |  |  |  |  |  |  |  |  |
| 2 |  | Attentional regulation | .43^**^ | 1 |  |  |  |  |  |  |  |  |  |  |  |  |  |  |  |  |  |  |  |  |  |  |  |
| 3 |  | Sleep problems | -.07^**^ | -.05^*^ | 1 |  |  |  |  |  |  |  |  |  |  |  |  |  |  |  |  |  |  |  |  |  |  |
| 4 |  | Receptive vocabulary | -.00 | .09^**^ | .03 | 1 |  |  |  |  |  |  |  |  |  |  |  |  |  |  |  |  |  |  |  |  |  |
| 5 |  | Peer problems | -.38^**^ | -.38^**^ | .02 | -.01 | 1 |  |  |  |  |  |  |  |  |  |  |  |  |  |  |  |  |  |  |  |  |
| 6 |  | Maternal consistency | .08^**^ | .11^**^ | -.10^**^ | .13^**^ | -.05^*^ | 1 |  |  |  |  |  |  |  |  |  |  |  |  |  |  |  |  |  |  |  |
| 7 |  | Paternal consistency | .09^**^ | .07^*^ | -.08^**^ | .05^*^ | -.03 | .29^**^ | 1 |  |  |  |  |  |  |  |  |  |  |  |  |  |  |  |  |  |  |
| 8 |  | Maternal warmth | .09^**^ | .07^**^ | .01 | -.00 | -.02 | .14^**^ | .08^**^ | 1 |  |  |  |  |  |  |  |  |  |  |  |  |  |  |  |  |  |
| 9 |  | Paternal warmth | .11^**^ | .07^*^ | -.01 | -.01 | -.09^**^ | .03 | .16^**^ | .21^**^ | 1 |  |  |  |  |  |  |  |  |  |  |  |  |  |  |  |  |
| 10 |  | Maternal anger | -.22^**^ | -.21^**^ | .14^**^ | -.05^*^ | .09^**^ | -.39^**^ | -.18^**^ | -.32^**^ | -.11^**^ | 1 |  |  |  |  |  |  |  |  |  |  |  |  |  |  |  |
| 11 |  | Paternal anger | -.17^**^ | -.14^**^ | .06^*^ | -.04 | .08^**^ | -.13^**^ | -.45^**^ | -.14^**^ | -.26^**^ | .35^**^ | 1 |  |  |  |  |  |  |  |  |  |  |  |  |  |  |
| 12 |  | Remoteness | -.01 | -.02 | -.01 | -.10^**^ | .02 | -.01 | .00 | -.04 | -.04 | .02 | .00 | 1 |  |  |  |  |  |  |  |  |  |  |  |  |  |
| 13 |  | Government school | .02 | -.02 | -.01 | -.02 | .02 | -.04 | -.04 | -.00 | -.05 | .02 | .03 | .03 | 1 |  |  |  |  |  |  |  |  |  |  |  |  |
| 14 |  | School enrolments | .00 | -.00 | -.02 | -.05^*^ | .04 | -.00 | -.02 | .03 | -.02 | .00 | .01 | -.17^**^ | -.09^**^ | 1 |  |  |  |  |  |  |  |  |  |  |  |
| 15 |  | Learning support | .02 | .01 | -.05 | .02 | -.01 | -.02 | .03 | .01 | .02 | -.02 | -.02 | -.01 | -.12^**^ | .12^**^ | 1 |  |  |  |  |  |  |  |  |  |  |
| 16 |  | Teacher view on work environment | .14^**^ | .07^**^ | -.0 | .06^*^ | -.09^**^ | .01 | .02 | .01 | -.02 | -.05 | -.03 | -.04 | -.04 | -.05^*^ | .03 | 1 |  |  |  |  |  |  |  |  |  |
| 17 |  | Teacher view on behaviour | .09^**^ | .03 | -.02 | .02 | -.08^**^ | .00 | -.03 | .00 | -.01 | -.04 | -.04 | -.01 | .01 | -.06^*^ | .04 | .54^**^ | 1 |  |  |  |  |  |  |  |  |
| 18 |  | Teacher qualification level | -.04 | -.03 | -.04 | .01 | -.01 | .02 | -.02 | -.03 | -.02 | .01 | .08^**^ | -.10^**^ | -.01 | .03 | -.01 | -.03 | -.03 | 1 |  |  |  |  |  |  |  |
| 19 |  | Teacher early childhood qualified | -.02 | -.01 | .00 | -.03 | .02 | -.01 | .01 | -.00 | -.03 | .02 | -.01 | .10^**^ | -.01 | .05 | .03 | .00 | -.02 | -.03 | 1 |  |  |  |  |  |  |
| 20 |  | Teacher self-efficacy | .09^**^ | .09^**^ | -.02 | .03 | -.09^**^ | .01 | .01 | .01 | .03 | -.02 | .00 | -.05 | .05 | .03 | -.02 | .30^**^ | .25^**^ | .01 | -.07^**^ | 1 |  |  |  |  |  |
| 21 |  | Parent school engagement | .09^**^ | .18^**^ | -.02 | .17^**^ | -.15^**^ | .14^**^ | .11^**^ | .11^**^ | .08^**^ | -.10^**^ | -.06 | .01 | -.02 | -.02 | .04 | .04 | -.00 | .02 | .03 | .05^*^ | 1 |  |  |  |  |
| 22 |  | Teacher-child closeness | .24^**^ | .29^**^ | -.00 | .02 | -.22^**^ | .06^*^ | .04 | .07^**^ | .04 | -.08^**^ | -.09^**^ | .01 | .01 | .01 | -.01 | .09^**^ | .06^*^ | -.04 | .02 | .21^**^ | .13^**^ | 1 |  |  |  |
| 23 |  | Child liking of teacher | .10^**^ | .12^**^ | -.01 | -.02 | -.09^**^ | .06^**^ | .05 | .06^**^ | .03 | -.11^**^ | -.09^**^ | .02 | -.03 | .04 | .00 | -.01 | -.05^*^ | -.03 | -.02 | .02 | .07^**^ | .16^**^ | 1 |  |  |
| 24 |  | Child school liking - emotional | .14^**^ | .12^**^ | -.02 | -.06^**^ | -.05^*^ | .07 | .02 | .10^**^ | .02 | -.12^**^ | -.08^**^ | -.01 | -.01 | .01 | .03 | -.02 | -.04 | .00 | -.02 | .00 | .04 | .11^**^ | .48^**^ | 1 |  |
| 25 |  | Child school liking - academic | .17^**^ | .23^**^ | -.01 | -.05^*^ | -.08^**^ | .05^*^ | .02 | .08^**^ | .04 | -.11^**^ | -.08^**^ | .01 | .01 | .02 | .04 | .04 | .05 | -.06^*^ | .00 | .02 | .03 | .12^**^ | .42^**^ | .52^**^ | 1 |
|  |  | Note: * = significant at p <.05; ** p < .01 |  |  |  |  |  |  |  |  |  |  |  |  |  |  |  |  |  |  |  |  |  |  |  |  |  |
