# Supplementary material for: Academic resilience from school entry to third grade: Child, parenting, and school factors associated with closing competency gaps
Source: PLoS One. 2022 Nov 30;17(11):e0277551. doi: 10.1371/journal.pone.0277551 (PMC9710847; doi:10.1371/journal.pone.0277551)
Supplement: S3 Appendix — (DOCX) [file pone.0277551.s003.docx]

**Appendix C**

# Paper: *Academic resilience from school entry to Grade 3: Child, parenting, and school factors*

**Formula for the calculation of predicted scores, demarcating academically resilient verses ongoing vulnerable children**

Analyses for this research question focussed only on children identified as academically vulnerable in the preliminary analysis described above. That is, children achieving the bottom 50% of scores on school entry language / cognitive developmental status (*n* = 781). Predicted scores for each domain of Grade 3 academic achievement were calculated using the following formula:

P_Achieve_ = M_Top50LG_ – (*β* x SD)

P_Achieve_ is the predicted score for the relevant achievement domain (reading or numeracy). *M*_Top50LG_ is the mean achievement score of the student group considered not vulnerable on school entry (i.e. those in the top 50% of scores in the language/cognition domain of the AEDC, who were ‘strong in Prep’). *β* is the standardized (with respect to the dependent variable of reading or numeracy score) regression coefficient that explains the expected standard deviation difference in academic domain scores for each of the three developmentally vulnerable groups (score ranges in the bottom 10%, 10 to 25%, and 25 to 50%) compared to the not vulnerable group (see Table A1). SD is the standard deviation of the mean of the overall sample scores for each achievement domain.

For example, the calculation of predicted Grade 3 reading achievement score for children in the bottom 10% of language/cognition development scores in their first year of school is:

P_Achieve_ = M_Top50LG_ + (*β* x SD)

P_Achieve_ = 467.98 + (-1.10 x 83.94)

P_Achieve_ = 375.65

Table C1 summarises the predicted score values obtained for reading and numeracy achievement using this formula.

**Table C1** Predicted score values using above formula

| **AEDC percentile group** | **Beta for reading** | **Beta for numeracy** | **Beta x SD (83.94) reading** | **Beta x SD (69.64) numeracy** | **PAchieve reading (predicted reading score)** | **PAchieve numeracy (predicted numeracy score)** |
| --- | --- | --- | --- | --- | --- | --- |
| Bottom 10% of the AEDC | -1.10 | -1.20 | -92.33 | -83.57 | 375.65 | 349.98 |
| 10 to 25th percentile | -0.75 | -0.83 | -62.96 | -57.80 | 405.03 | 375.75 |
| 26th to 50th percentile | -0.44 | -0.42 | -36.93 | -29.25 | 431.05 | 404.30 |
