# Supplementary material for: Academic resilience from school entry to third grade: Child, parenting, and school factors associated with closing competency gaps
Source: PLoS One. 2022 Nov 30;17(11):e0277551. doi: 10.1371/journal.pone.0277551 (PMC9710847; doi:10.1371/journal.pone.0277551)
Supplement: S4 Appendix — (DOCX) [file pone.0277551.s004.docx]

**Appendix D**

# Paper: *Academic resilience from school entry to Grade 3: Child, parenting, and school factors associated with closing competency gaps.*

**Table D1** *Descriptive differences on mean scores between resilient and ongoing vulnerable groups on explanatory variables, including those not statistically significant and not shown in paper Table 3.*

|  | **Reading** | | **Numeracy** | |
| --- | --- | --- | --- | --- |
|  | Resilient | Ongoing vulnerable | Resilient | Ongoing vulnerable |
| *Child factors* |  |  |  |  |
| Attentional regulation | 18.96** | 16.71 | 18.89** | 16.62 |
| Emotional regulation | 21.44 | 21.60 |  |  |
| Sleep problems | 1.36 | 1.37 | 1.26** | 1.44 |
| Vocabulary | 75.21** | 72.28 | 74.61** | 72.52 |
| Peer problems | 5.96** | 6.32 | 6.00* | 6.31 |
| *Family factors* |  |  |  |  |
| Maternal consistency | 4.33* | 4.21 | 4.34** | 4.20 |
| Paternal consistency | 4.27** | 4.09 | 4.29** | 4.06 |
| Maternal anger | 1.71** | 1.84 | 1.70** | 1.87 |
| Paternal anger | 1.71 | 1.78 | 1.68** | 1.82 |
| Maternal warmth | 4.54 | 4.55 | 4.56 | 4.54 |
| Paternal warmth | 4.19 | 4.19 | 4.15 | 4.21 |
| *School factors* |  |  |  |  |
| Remoteness^ | .73** | .93 | .73** | .94 |
| Government school (%) | 68.4 | 69.1 | 64.8* | 71.9 |
| *School size* | 528.61 | 543.65 | 531.20 | 542.60 |
| Teacher has access to learning support in classroom (%) | 48.2 | 51.6 | 51.5 | 48.2 |
| Teacher-reported positive work environment | 4.28 | 4.26 | 4.28 | 4.26 |
| Whole-school behavior approach | 4.03 | 4.05 | 4.09 | 4.01 |
| Teacher level of education | 4.05 | 4.10 | 4.10 | 4.06 |
| Teacher trained in early childhood (%) | 21.0 | 25.7 | 21.9 | 25.3 |
| Teacher self-efficacy | 4.46 | 4.42 | 4.49* | 4.40 |
| Parent engagement (teacher reported) | 3.33* | 3.12 | 3.44** | 3.04 |
| Parent engagement (parent reported) | 16.75 | 16.45 | 16.60 | 16.54 |
| Teacher closeness to child | 4.26 | 4.26 | 4.27 | 4.26 |
| Child likes teacher | 2.69 | 2.71 | 2.68 | 2.73 |
| Child likes social-emotional aspects of school | 2.53 | 2.51 | 2.50 | 2.53 |
| Child likes academic aspects of school | 2.59 | 2.56 | 2.58 | 2.56 |

^ higher scores indicate a more remote area; # total enrolment; ** significantly different to the ongoing vulnerable group at p < .01; * significantly different at p < .05.
